# The Impact of NRF2 Inhibition on Drug-Induced Colon Cancer Cell Death and p53 Activity: A Pilot Study

**DOI:** 10.3390/biom12030461

**Published:** 2022-03-17

**Authors:** Alessia Garufi, Giuseppa Pistritto, Valerio D’Orazi, Mara Cirone, Gabriella D’Orazi

**Affiliations:** 1 Unit of Cellular Networks, Department of Research and Advanced Technologies, IRCCS Regina Elena National Cancer Institute, 00144 Rome, Italy; alessiagufi@yahoo.it; 2 Centralized Procedures Office, Italian Medicines Agency (AIFA), 00187 Rome, Italy; pistritto@med.uniroma2.it; 3 Department of Surgical Sciences, Sapienza University, 00185 Rome, Italy; valerio.dorazi@uniroma1.it; 4 Laboratory Affiliated to Pasteur Institute Italy Foundation Cenci Bolognetti, Department of Experimental Medicine, Sapienza University, 00185 Rome, Italy; mara.cirone@uniroma1.it; 5 Department of Neurosciences, Imaging and Clinical Sciences, University “G. D’Annunzio”, 66013 Chieti, Italy; 6 UniCamillus International Medical University in Rome, 00131 Rome, Italy

**Keywords:** NRF2, sulforaphane, TP53, colorectal carcinoma, ZnCl_2_ supplementation, apoptosis, DNA damage, unfolded protein response (UPR), CHOP

## Abstract

Nuclear factor erythroid 2 (NF-E2) p45-related factor 2 (NRF2) protein is the master regulator of oxidative stress, which is at the basis of various chronic diseases including cancer. Hyperactivation of NRF2 in already established cancers can promote cell proliferation and resistance to therapies, such as in colorectal cancer (CRC), one of the most lethal and prevalent malignancies in industrialized countries with limited patient overall survival due to its escape mechanisms in both chemo- and targeted therapies. In this study, we generated stable NRF2 knockout colon cancer cells (NRF2-Cas9) to investigate the cell response to chemotherapeutic drugs with regard to p53 oncosuppressor, whose inhibition we previously showed to correlate with NRF2 pathway activation. Here, we found that NRF2 activation by sulforaphane (SFN) reduced cisplatin (CDDP)-induced cell death only in NRF2-proficient cells (NRF2-ctr) compared to NRF2-Cas9 cells. Mechanistically, we found that NRF2 activation protected NRF2-ctr cells from the drug-induced DNA damage and the apoptotic function of the unfolded protein response (UPR), in correlation with reduction of p53 activity, effects that were not observed in NRF2-Cas9 cells. Finally, we found that ZnCl_2_ supplementation rescued the cisplatin cytotoxic effects, as it impaired NRF2 activation, restoring p53 activity. These findings highlight NRF2′s key role in neutralizing the cytotoxic effects of chemotherapeutic drugs in correlation with reduced DNA damage and p53 activity. They also suggest that NRF2 inhibition could be a useful strategy for efficient anticancer chemotherapy and support the use of ZnCl_2_ to inhibit NRF2 pathway in combination therapies.

## 1. Introduction

Colorectal cancer (CRC) ranks as the second most lethal cancer and the third most prevalent malignant tumor worldwide [1]. The basis of CRC treatment consists of surgery, targeted therapy, neoadjuvant radiotherapy and adjuvant chemotherapy. Unfortunately, drug-resistance remains one of the major reasons for the low survival rates of CRC patients [2]. A better understanding of the mechanisms leading to the intrinsic and acquired resistance to therapies will be a great asset for prognostic and therapeutic purpose in CRC. Among the molecular pathways altered in CRC, aberrant hyperactivation of Nuclear factor erythroid 2 (NF-E2) p45-related factor 2 (NRF2) can occur and correlates with poor patients’ prognosis [3,4]. NRF2 is the master regulator of oxidative stress and is often upregulated in solid cancers, promoting cell proliferation and resistance to therapy [3,5]. NRF2 stabilization/activation can be achieved by inhibition of Kelch-like ECH associated protein 1 (Keap1)-associated E3 ubiquitin ligase activity after oxidative or electrophilic stress [6]. Alternatively, NRF2 activation is attained by p62/sequestome 1 (SQSTM1)/Keap1 or p21^Cip1/WAF1^/Keap1 interaction [7,8], as well as by inactivating mutations on the *KEAP1* gene or gain-of-function mutations on the NRF2 gene (*NFE2L2*) [3], although genetic alterations of *KEAP1* or *NFE2L2* are rare in CRC. In all cases, NRF2 activation induces the transcription of antioxidant or antiapoptotic target genes (such as heme oxygenase 1-HO-1) that protect cells from oxidative stress, which is at the basis of many chronic diseases, including cancer [9,10]. NRF2 transient activation is considered to be mainly cytoprotective during the first phases of cancerogenesis, while NRF2 persistent activation may act as a driver of cancer progression, metastasis, and resistance to therapies [3]. For these reasons, NRF2 has become an attractive molecule to be targeted in order to enhance the efficacy of the current cancer treatments.

The tumor suppressor p53 is activated in response to several stresses such as DNA damage to induce the transcription of target genes involved in anticancer functions, including cell cycle arrest, apoptotic cell death, senescence, etc. [11]. The DNA damage can be detected by phosphorylation of H2AX in Ser139, generating γH2AX, that occurs mainly in response to double-strand brakes (DSB) [12]. For its key role as sensor of DNA damage, p53 undergoes inactivation, both at genetic and/or protein level, in almost all tumors [13], and its inactivation predisposes to cancer onset progression and resistance to therapies [14,15]. In this regard, we have demonstrated that ZnCl_2_ supplementation can both reactivate mutant p53, restoring cancer cell chemosensitivity, and improve wild-type p53 function [16,17]. Intriguingly, we recently found that ZnCl_2_ supplementation may enhance the pro-death function of the unfolded protein response (UPR) through the activation of the apoptotic marker C/EBP homologous protein (CHOP) [18], increasing the DNA damage and p53 function [19], encouraging its use in anticancer combination therapies. It is indeed emerging that endoplasmic reticulum (ER) stress and UPR activation can have an impact on DNA damage given the role of UPR sensors in regulating the expression of molecules involved in DNA repair [20]. In line with this evidence, we have also recently shown that the inhibition of ATF6 can activate wtp53 and even downregulate mutp53 in colon cancer cells [21]. Taking advantage of our previous findings where activation of the NRF2 pathway by high glucose or curcumin compounds reduces cancer cell sensitivity to anticancer agents in parallel with impairment of the p53 activity [15,22,23,24,25], the aim of this study was to investigate the cell response to chemotherapeutic drugs, with regard to p53 activity, by generating NRF2-null colon cancer cells with the CRISPR-Cas9 technology. We used sulforaphane (1-isothiocyanato-4-methylsulfynbutane) (SFN), a compound derived from cruciferous vegetables, as one of the most potent inducers of NRF2 (by directly liberating NRF2 from Keap1-dependent degradation) [26] to investigate the colon cancer cell response to cisplatin (CDDP) alone or in combination with ZnCl_2_ supplementation.

## 2. Materials and Methods

### 2.1. Cell Culture and Reagents

Human colon cancer HCT116, HCT116-p53^−/−^ (kindly provided by Prof. Bert Volgelstein, Johns Hopkins University, Baltimore, MD, USA), colon cancer RKO and the human lung cancer H1299 (p53 null) cells were maintained in Dulbecco’s modified Eagle’s medium (DMEM) (Life Technologies-Invitrogen, Eggenstein, Germany), containing 10% heat-inactivated Foetal Bovine Serum (FBS) (Corning, NY, USA) and L-glutamine/streptomycin (100 µg/mL) (Corning, NY, USA) in a culture incubator with 5% CO_2_ at 37 °C in humidified atmosphere. They underwent routine testing to ensure that they were mycoplasm negative. The activator of NRF2, that is, D,L-Sulforaphane (1-isothiocyanato-4-methylsulfinylbutane (SFN) (Sigma-Aldrich, St. Louise, MO, USA) [26] was dissolved in DMSO and used at various concentrations (1, 2, 5 µM) for 24 h, alone or in combination with drugs; chemotherapeutic drug cisplatin (CDDP) (Pharmachemie BV, The Netherlands) was added to the cell culture at 5 μg/mL, as previously reported [22]; and ZnCl_2_ (Sigma-Aldrich) was dissolved in dH_2_O_2_ and used at 100 μM [27].

### 2.2. Generation of CRISPR/Cas9-Based NRF2- Knockout HCT116 Cells (NRF2 KO Cells)

NRF2 CRISPR/Cas9 KO plasmid (sc-400017) and NRF2 Homology Directed Repair (HDR) plasmid (sc-400017) were purchased from Santa Cruz Biotechnology, USA. The NRF2 CRISPR/Cas9 KO plasmid consists of a pool of 3 plasmids, each encoding the Cas9 nuclease and a target-specific 20 nt guide RNA designed for maximum knockout efficiency. The NRF2 HDR plasmid integrates puromycin resistance genes into cut sites which are generated by the NRF2 CRISPR/Cas9 KO plasmid. NRF2 CRISPR/Cas9 KO and NRF2 HDR plasmids were co-transfected into HCT116 colon cancer cells, using UltraCruz Transfection Reagent (Santa Cruz Biotechnology, Dallas, TX, USA). GFP region was added in NRF2 CRISPR/Cas9 KO and RFP was added in NRF2 HDR plasmid to visualize the transfection efficiency. Therefore, both plasmids transfected cells are able to have GFP and RFP intensities. After transfection, cells were treated with puromycin (Santa Cruz Biotechnology, Dallas, TX, USA) for 48 h, and selected by single-cell dilution in 96-well plates. According to NRF2 downregulation results, clones 1 and 2 were selected. Clone 2 was used in this study to evaluate the mechanism of NRF2 deficiency (Figure S1).

### 2.3. Cell Viability Assays

Cells were plated in six-well plates using three to five replicates. The day after plating, cells were treated with CDDP (5 μg/mL) for 24 h with or without cotreatment with SFN (2 µM) and/or ZnCl_2_ (100 μM). After treatments, cells (both floating and adherent ones) were collected and stained with Trypan blue (Sigma-Aldrich, Dorset, UK). Cell viability was assessed by counting blue (dead)/total cells with a Neubauer hemocytometer using light microscopy.

### 2.4. Proliferation Assay (XTT)

The Cell Proliferation II kit (Roche Diagnostic) is based on the ability of viable cells to cleave the tetrazolium ring of 2,3-bis(2-methoxy-4-nitro-5-sulfophenyl)-2H-tetrazolium-5-carboxyanilide (XTT) inner salt, yielding orange formazan crystals soluble in acqueous solutions. Briefly, cells were seeded in 96-well culture plates (5 × 10^3^ cells/well) and the day after plating treated with SFN for the indicated time and doses. After treatment and following the manufacturer’s instructions, XTT was added and cells incubated for 4 h before stopping the formazan formation adding the solubilization solution. The absorbance was recorded on a microplate reader at a wavelength of 492 nM.

### 2.5. Western Blotting

Cells were lysed in lysis buffer (50 mM Tris–HCl, pH 7.5, 150 mM NaCl, 5 mM EDTA, 150 mM KCl, 1 mM dithiothreitol and 1% Nonidet P-40) (all from Sigma- Aldrich, Dorset, UK) containing protease inhibitors (CompleteTM, Mini Protease Inhibitor Cocktail, Merck, Life Science S.r.l., Milan, Italy), sonicated and then centrifuged at 4 °C for 20 min. Proteins were separated by loading 10–30 ug of total cell lysates on denaturing 8–15% SDS-PAGE (polyacrylamide gel electrophoresis) gels (Bio-Rad, Hercules, CA, USA), following semidry blotting to polyvinylidene difluoride (PVDF) membranes (Immobilon-P, Merk-Millipore, Milan, Italy). Membranes were blocked in Tris buffered saline containing 0.1% Tween 20 (TBS) and 3% BSA (Merck-Sigma-Aldrich, Darmstadt, Hesse, Germany) before probing with the primary antibodies and then with the appropriate secondary antibodies coupled to horseradish peroxidase (HRP) (Bio-Rad Laboratories, Segrate, Italy). Enzymatic signal was visualized by chemiluminescence (ECL Detection system, Amersham GE Healthcare, Milan, Italy).

The antibodies used for Western blotting analyses were: mouse monoclonal anti-HO-1, mouse monoclonal p53 (DO1), rabbit polyclonal phospho-Ser46, rabbit polyclonal p21, mouse monoclonal anti-PARP (poly ADP-ribose polymerase 1, cleaved form), mouse monoclonal anti-phospho-Histone H2AX (Ser139) (all from, Santa Cruz Biotechnology, Dallas, TX, USA), rabbit polyclonal anti-NRF2 (Abcam, Cambridge, UK), rabbit polyclonal anti-CHOP (GADD153) (Proteintech, Rosemont, IL, USA), and mouse monoclonal β-actin (Calbiochem, San Diego, CA, USA). Densitometry was performed on ECL results with ImageJ software (https://imagej.nih.gov, accessed on 10 February 2022) and relative band intensity normalized to β-actin and plotted as protein expression/β-actin ratio.

### 2.6. RNA Extraction and Semiquantitative Reverse Transcription (RT)-Polymerase Chain Reaction (PCR) Analysis

Total RNA extraction was performed by using TRIzol Reagent (Thermo Fisher Scientific, Walthman, MA, USA); cDNA was synthesized by using an MuLV reverse transcriptase kit (Applied Biosystems, Foster City, CA, USA); semiquantitative Reverse-Transcribed (RT)-PCR was carried out with 2 μL cDNA reaction and genes-specific oligonucleotides under conditions of linear amplification, by using Hot-Master Taq polymerase (Thermo Fisher Scientific, Walthman, MA, USA). PCR products were run on a 2% agarose gel and visualized with GelRed Nucleic Acid gel stain (Biotium, San Francisco, CA, USA). The housekeeping 28S gene, used as internal standard, was amplified from the same cDNA reaction mixture. Densitometric analysis was applied to quantify mRNA levels compared to control gene expression.

### 2.7. Statistical Analysis

Results are expressed as values of mean ± standard deviation (S.D.). Statistical significance was determined using Student’s *t*-tests for two-sample comparison and one-way ANOVA analysis for two or more samples comparison. Difference was considered statistically significant when the *p*-value was ≤0.05.

## 3. Results

### 3.1. Sulforaphane Reduces the Cisplatin (CDDP)-Induced Colon CancerCcell Death

**Figure 1:**
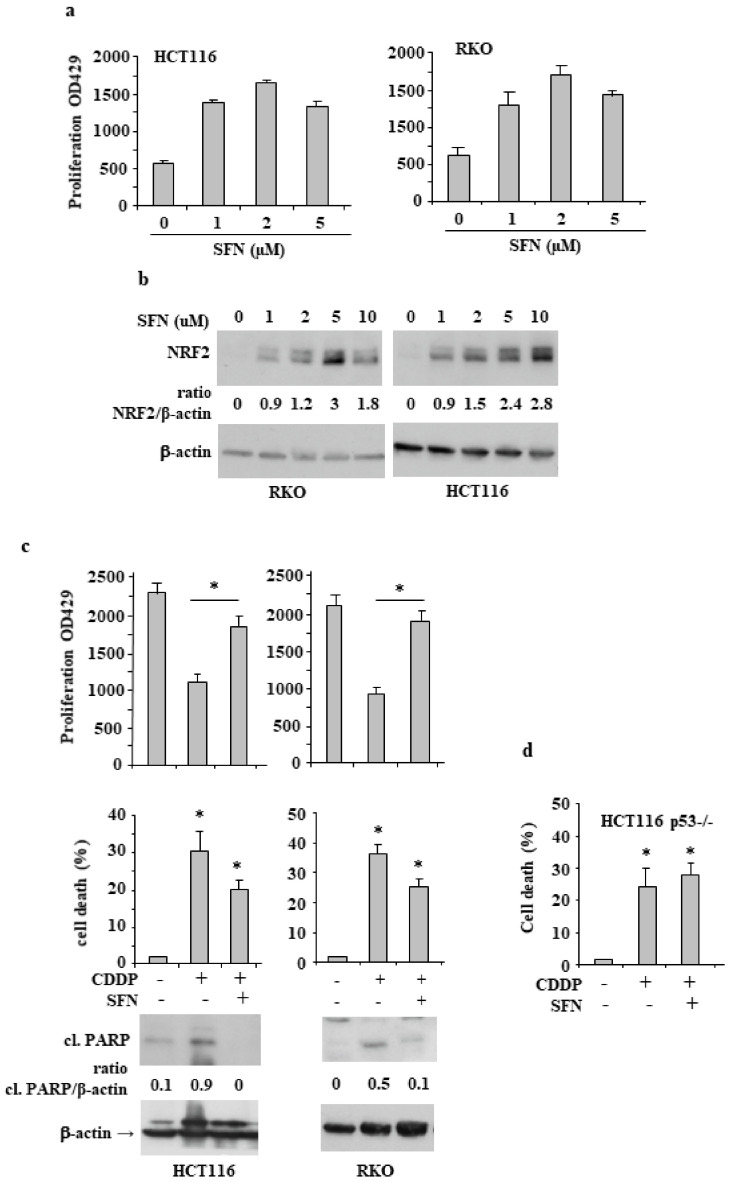
Sulforaphane (SFN) induces NRF2 protein levels and reduces drug-induced cell death. (**a**) HCT116 and RKO cells were exposed to increasing doses of SFN for 24 h, and cell viability was assessed by XTT assay. The histograms represent the mean plus S.D. from three independent experiments. (**b**) Cells treated as in (**a**) were analyzed by Western blot for NRF2 expression levels. Actin was used as protein loading control. The ratio of NRF2 levels vs. β-actin, following densitometric analysis using ImageJ software, is shown. (**c**) In the upper panel, HCT116 and RKO cell viability was measured by XTT assay at 492 nM and cell death was measured by Trypan blue staining after treatment with cisplatin (CDDP) (5 µg/mL) alone or in combination with SFN (2 µM) for 24 h. The results are expressed as cell death percentage ± S.D. In the lower panels, the expression levels of PARP cleavage (cl.) were assessed by Western blot. Actin was used as protein loading control and the ratio of cl.PARP vs. β-actin, is reported. (**d**) Cell viability, as measured by Trypan blue staining, of HCT116-p53^−/−^ cells treated as in (**c**) The results are expressed as cell death percentage ± S.D. * *p* ≤ 0.01.

We first looked at the influence of sulforaphane (SFN), as NRF2 inducer [26], on cancer cell proliferation. We performed a proliferation XTT assay after treating RKO and HCT116 colon cancer cells with increasing doses of SFN (1, 2, 5 µM) for 24 h. As shown in Figure 1a, SFN increased cell proliferation in both cell lines which correlated with concomitant NRF2 protein stability (Figure 1b). Then, we asked whether SFN could affect drug-induced cell death. To this aim, colon cancer cells were incubated with cisplatin (CDDP) at the dose of 5 μg/mL that we previously showed to induce cell death [27], with or without SFN cotreatment. The results show that the cell proliferation reduced by CDDP (Figure 1c, upper panels) and the cell death induced by CDDP (Figure 1c, middle panels) were significantly counteracted by SFN cotreatment; in agreement, apoptotic PARP cleavage induced by CDDP was reduced by SFN cotreatment (Figure 1c, lower panels). Since p53 is a key molecule in inducing cell death in response to chemotherapeutic drugs, we evaluated the role of p53 in CDDP/SFN treated cancer cells by using HCT116 p53^−/−^ cells. We found that the CDDP-induced cell death was not reduced by SFN cotreatment in p53^−/−^ cells (Figure 1d), suggesting a role for p53 in SFN reduction of drug cytotoxicity.

### 3.2. NRF2 Is Involved in Reduction of CDDP Citotoxycity

**Figure 2:**
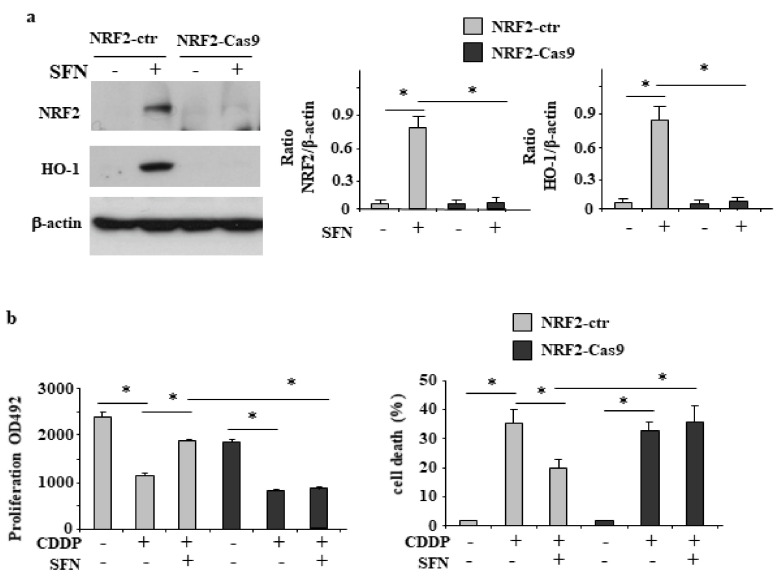
NRF2 is involved in SFN-induced inhibition of CDDP cytotoxicity. (**a**) NRF2-proficient (NRF2-ctr) and NRF2-KO (NRF2-Cas9) cells were treated with SFN (2 µM) for 8 h and NRF2 and HO-1 protein levels analyzed by Western blot. Actin was used as protein loading control. Densitometric analysis of NRF2/β-actin and HO-1/β-actin is reported in the right panels. * *p* ≤ 0.01. (**b**) NRF2-ctr and NRF2-Cas9 cell proliferation (left panel) was measured by XTT assay and cell viability (right panel) was measured by Trypan blue staining after treatment with cisplatin (CDDP) (5 µg/mL) alone or in combination with SFN (2 µM) for 24 h. The results are expressed as cell death percentage ± S.D. * *p* ≤ 0.01.

Since NRF2 is an important prosurvival molecule, we attempted to inhibit it by generating NRF2-null HCT116 cells with the CRISPR/Cas9 technology (Figure S1). As shown in Figure 2a, SFN failed to induce NRF2 and its target HO-1 in NRF2-Cas9 cells, compared to the NRF2 proficient (NRF2-ctr) cells, confirming the loss of NRF2 activity in NRF2-Cas9 cells. Then, we checked the role of NRF2 in response to drugs by treating NRF2-ctr and NRF2-Cas9 cells with CDDP, with or without SFN cotreatment. The results show that the cell proliferation, as assessed by the MTT assay (Figure 2b, left panel), reduced by CDDP, and the cell death (Figure 2b, right panel) induced by CDDP in both cell lines, were significantly counteracted by SFN only in NRF2-ctr cells, compared to the NRF2-Cas9 ones, demonstrating the NRF2 key role in counteracting drug cytotoxyc effect.

### 3.3. NRF2 Inhibition Restores CDDP-Induced p53 Activity Impaired by SFN

**Figure 3:**
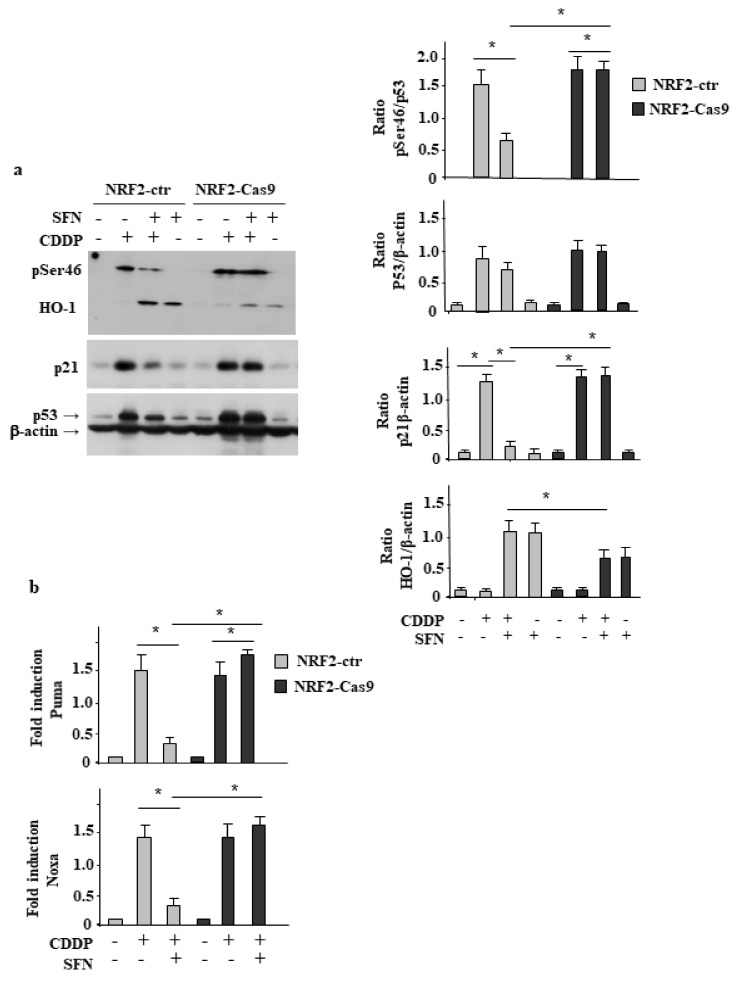
NRF2 inhibition restores CDDP-induced p53 activity impaired by SFN. (**a**) NRF2-ctr and NRF2-Cas9 cells were treated with cisplatin (CDDP) (5 µg/mL) alone or in combination with SFN (2 µM) for 24 h before assessing the phospho (p) Ser46, p53 and HO-1 levels by Western blot. Actin was used as protein-loading control. Densitometric analysis of pSer46/p53, p53/β-actin and HO-1/β-actin is reported in the right panels. * *p* ≤ 0.01. (**b**) Total mRNA was extracted from NRF2-ctr and NRF2-Cas9 cells untreated or treated as in (**a**). The indicated gene expression was assayed by semiquantitative RT-PCR. The histograms represent the mean plus S.D. of three independent experiments. Densitometric analysis using ImageJ software was applied to calculate the gene/28S ratio. * *p* ≤ 0.01.

Then, we evaluated the involvement of p53 in our cell system. We found that p53 activation following CDD treatment, seen as p53Ser46 phosphorylation [27] and p21 protein levels, was efficiently inhibited by SFN only in NRF2-proficient cells (NRF2-ctr), compared to the NRF2-Cas9 cells (Figure 3a), and this outcome correlated with the cell death results shown above (Figure 2b). In agreement, HO-1 levels, induced by SFN remained high in the CDDP/SFN combination in NRF2-ctr cells, in line with the HO-1 role in chemoresistance [28]. Then, p53 transcription activity was evaluated by RT-PCR of mRNA levels. The results show that drug-induced upregulation of p53 apoptotic target genes Puma and Noxa was strongly impaired by SFN only in NRF2-ctr cells, while it was not affected in NRF2-Cas9 ones (Figure 3b). Altogether, these findings indicate that NRF2 inhibition could maintain an efficient p53 activity and cell death in response to drug that, conversely, were strongly reduced in the presence of an active NRF2 pathway.

### 3.4. NRF2 Activation Impairs the CDDP-Induced DNA Damage

**Figure 4:**
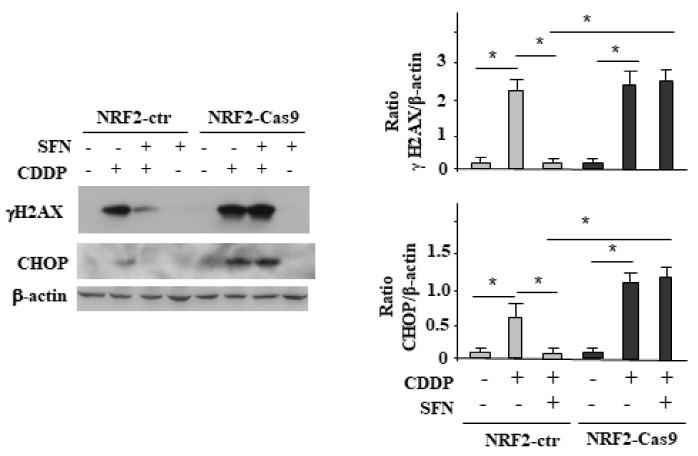
NRF2 activation impairs the CDDP-induced DNA damage. NRF2-ctr and NRF2-Cas9 cells were treated with CDDP (5 µg/mL) alone or in combination with SFN (2 µM) for 24 h. The indicated proteins’ expression was analyzed by Western blot and the densitometric analyses reported in the right panels with S.D. Actin was used as protein loading control. * *p* ≤ 0.01.

In order to find an explanation to the reduction of p53 activation in the presence of NRF2, we evaluated the DNA damage response and the markers of the unfolded protein response (UPR). To this aim, we exposed NRF2-ctr and NRF2-Cas9 cells to CDDP, with or without SFN co-treatment and assessed by immunoblotting the phosphorylation of H2AX in Ser139, generating γH2AX, that occurs in general in response to double-strand brakes (DSB) [12]. As shown in Figure 4, the CDDP-induced γH2AX levels in both cell lines were strongly reduced by SFN only in NRF2-ctr cells, compared to the NRF2-Cas9 ones. In agreement, the CDDP-induced upregulation of the promortem unfolded protein response (UPR) molecule CHOP, in both cell lines, was impaired by SFN only in NRF2-ctr cells, compared to the NRF2-Cas9 ones. These findings suggest that NRF2 pathway was protecting cells from CDDP-induced cell death by reducing DNA damage and the proapoptotic function of UPR. Conversely, NRF2 inhibition could be a promising strategy for maintaining the cytotoxic effect of the anticancer drugs.

### 3.5. ZnCl_2_ Supplementation Rescues p53 Activity Inhibited by SFN

**Figure 5:**
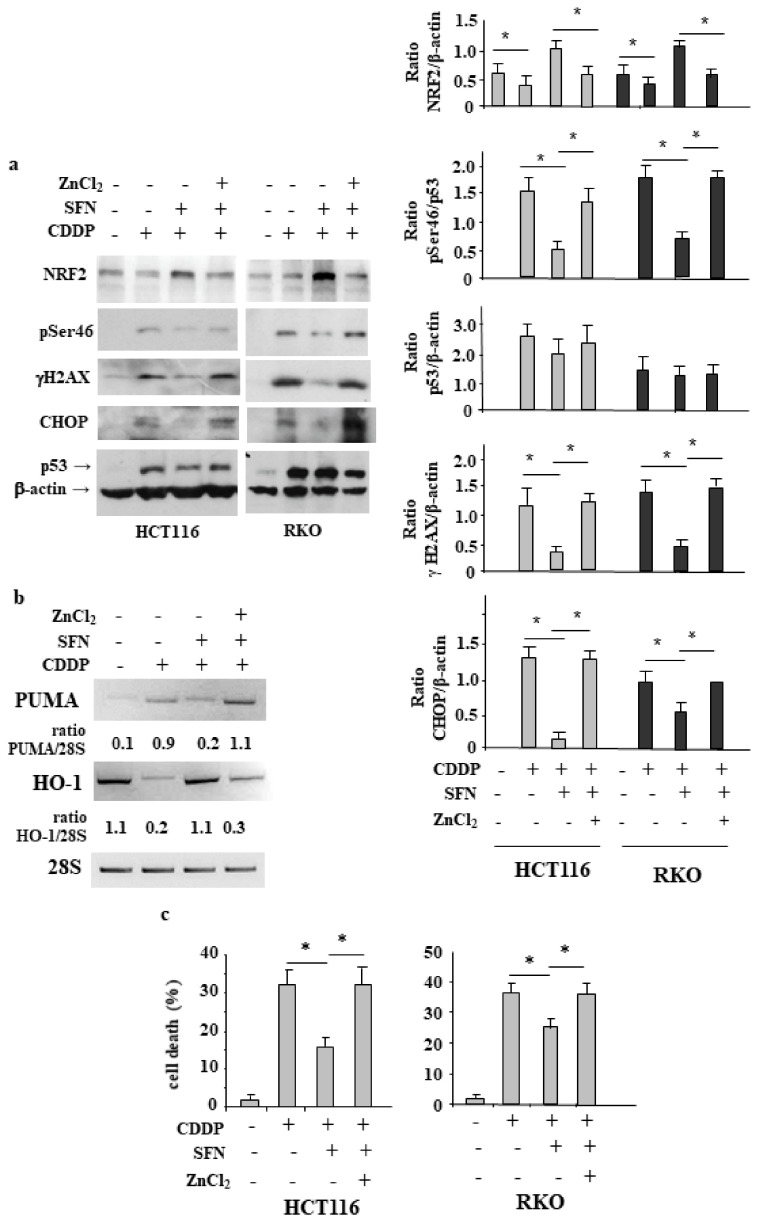
ZnCl_2_ supplementation enhances DNA damage and rescues p53 activity, inhibited by SFN. (**a**) HCT116 and RKO cells were treated with CDDP (5 µg/mL) alone or in combination with SFN (2 µM) and ZnCl_2_ (100 μM). The expression level of the indicated proteins was analyzed by Western blot. Actin was used as protein loading control. Densitometric analyses was performed and reported as histogram in the right panels, plus S.D. * *p* ≤ 0.01. (**b**) RT-PCR analysis of total mRNA extracted from HCT116 cells treated as in (**a**). Densitometric analysis using ImageJ software was applied to calculate the PUMA/28S ratio. (**c**) HCT116 and RKO cell viability was measured by Trypan blue staining after treatment with cisplatin (CDDP) (5 µg/mL) alone or in combination with SFN (2 µM) and ZnCl_2_ (100 μM) for 24 h. The results are expressed as cell death percentage ± S.D. * *p* ≤ 0.01.

In previous studies we have shown that ZnCl_2_ supplementation can reactivate p53 oncosuppressor function, inhibited by different conditions, including high antioxidant response [16,17,27,29,30,31]. Therefore, here we aimed to evaluate whether ZnCl_2_ supplementation could restore the CDDP-induced cell death and p53 activity, following NRF2 activation, in NRF2-proficient cells. As shown in Figure 5a, we found that p53Ser46 activation, following CDD treatment and inhibited by SFN cotreatment, was restored by ZnCl_2_ supplementation and that such treatment prevented NRF2 up-regulation. Moreover, the reduction of γH2AX and CHOP levels in CDDP/SNF treatment, compared to CDDP alone, was counteracted by ZnCl_2_, taking back their levels to those achieved in CDDP treatment. These data suggest the reestablishment of CDDP-induced DNA damage by ZnCl_2_, which also correlated with the reestablishment of p53 activation and inhibition of NRF2. At molecular level, CDDP-induced p53 target gene expression, inhibited by SFN, was rescued by ZnCl_2_ supplementation and, in an opposite way, the high expression levels of HO-1 in CDDP/SFN treatment were counteracted by ZnCl_2_ supplementation (Figure 5b). Finally, cell viability assay shows that the CDDP-induced cell death, reduced by SFN, was efficiently rescued by ZnCl_2_ (Figure 5c). These data suggest that the pathways triggered by NRF2 reduced drug cytotoxicity and p53 activity; however, this outcome was counterbalanced by ZnCl_2_ supplementation, although the molecular mechanisms need to be elucidated.

## 4. Discussion

**Figure 6:**
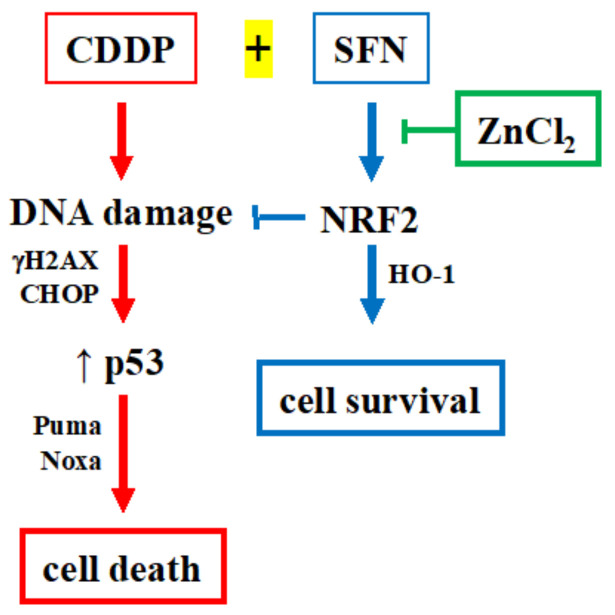
Proposed model for NRF2 role in cancer cell chemosensitivity and p53 activity. Hyperactivation of NRF2 (by SFN) counteracts the CDDP-induced DNA damage, impairing the p53 activity and reducing cell death; ZnCl_2_ supplementation counteracts the effect of SFN/NRF2 rescuing the DNA damage, p53 activity, and cell death, induced by CDDP.

This study deals with the involvement of NRF2 as prosurvival molecule that can reduce the cytotoxic effect of drug treatment of cancer cells. By generating stable NRF2 knockout HCT116 colon cancer cells (NRF2-Cas9) we found that NRF2 activation with sulforaphane (SFN), one of the most potent NRF2 inducer [26], on one hand reduced cisplatin (CDDP)-induced cancer cell death in NRF2-proficient cells, while did not affect it in NRF2-Cas9 cells, highlighting the key role for NRF2 in inhibiting cancer cell response to anticancer drugs. Mechanistically, we found that NRF2 activation, leading to high levels of HO-1, protected NRF2-proficient cells from the drug-induced DNA damage and the pro-apoptotic function of the unfolded protein response (UPR), and also reduced p53 apoptotic activity, all mechanisms that were not inhibited in NRF2-Cas9 cells that underwent an even more efficient cell death compared to the NRF2-proficient cells. In the attempt to block the NRF2 prosurvival function, we found that ZnCl_2_ supplementation rescued the CDDP cytotoxic effects, impaired by NRF2 activation in NRF2-proficient cells, restoring the DNA damage, the UPR apoptotic function and the p53 apoptotic activity, proposing the model shown in Figure 6.

Resistance to chemotherapy is a major obstacle to successful anticancer treatments, including treatment of CRC, and can cause tumor recurrence (or relapse) and even invasion and metastasis [2,32]. Among the molecules implicated in cancer chemoresistance is NRF2, which is often upregulated in solid cancers promoting proliferation, chemoresistance and inhibition of apoptosis [3]. In normal cells, the NRF2-antioxidant stress transcriptional program plays an important role in tumor prevention. As reactive oxygen species (ROS) can induce DNA damage and promote gene mutation and genome instability, leading to tumor development, antioxidant proteins can be helpful in preventing cancer initiation by counteracting DNA damage [9], as demonstrated by genetic approach showing reduced cancer incidents in NRF2 wild-type but not knockout mice, following chemical carcinogenesis [33]. The NRF2 detoxifying activity is important in cancer prevention; however, cancer cells can hijack this protective mechanism to promote tumor progression and resistance to chemotherapy. By using a genetic approach, that is, a murine model of mutant K-Ras driven lung and pancreatic cancer, it has been demonstrated that NRF2 knockout reduces tumor proliferation and increases overall survival [34], leading to a new procancer activity for NRF2. In line with this concept, it has been found that upregulation of the NRF2 pathway in colorectal tumors correlates with a poor patient prognosis [4]. Among the NRF2 targets, Heme oxygenase 1 (HO-1) is considered one of the main effectors of antioxidant response [35]. Intriguingly, high levels of HO-1 have been found in various human tumors, inducing survival advantage, aggressiveness, and poor outcome, and in vitro and in vivo studies, including clinical data, have shown that the inhibition or silencing of HO-1 prevents this behavior [36].

Oncosuppressor p53 is considered as the guardian of the genome and its function is crucial to suppress DNA mutation and protect mammals from tumorigenesis. The extent of DNA damage can differentially activate p53 through diverse posttranslational modifications and dictate the cellular outcome between cell cycle arrest or apoptosis [37]. Under severe DNA damage, p53 undergoes phosphorylation at N-terminal serine 46 (Ser46), which is considered an important readout of p53 apoptotic activity [38,39], while, under low stress, p53 activity is shifted from apoptosis to cell cycle arrest, with abrogation of Ser46 phosphorylation, as we previously showed associated to the activation of the p53 proapoptotic kinase HIPK2 [40]. In this study we found that NRF2 activation correlated with the inhibition of phosphorylation of p53 Ser46 and with the reduction of CDDP-induced cell death, highlighting a link between NRF2 and HIPK2/p53 and the balance between cell death/cell survival in response to DNA damage. In line with this theory, it has been shown that, in high-stress conditions, high p53 levels suppress the NRF2-mediated cell survival pathway and induce cell apoptosis, while in low-stress conditions, low p53 levels upregulate the NRF2 pathway likely through p21, which stabilizes NRF2 [8,41]. Reduction of HIPK2 activity has been shown to impair the p53 apoptotic response [40,42], p21 has been shown to induce NRF2 in a noncanonical way [8] and NRF2 has been suggested to modulate HIPK2 function [43,44]. These findings suggest that unveiling the mechanisms that connect NRF2 to HIPK2/p53 pathway could be worthwhile in order to predict cancer cell response to anticancer therapies, since all three molecules are important for therapy response in cancer [3,11,45].

The search for new molecules that inhibit the NRF2 pathway to improve anticancer therapies have great therapeutic potential as supplementing traditional cancer therapies, although their use is not yet in clinical practice given their variable mechanisms of action and outcomes [46]. Here, ZnCl_2_ supplementation was found to efficiently inhibit the pathway triggered by NRF2 and restore DNA damage and apoptotic UPR in response to CDDP. Zinc ions play an important role in p53 biology [29] and we found here an additional function as an NRF2 inhibitor, likely by modulating the Keap1 activity [47] although this latter hypothesis needs to be clarified. From a translational point of view, given the low toxicity of zinc supplementation in vivo, its use in combination therapy would not be harmful for cancer patients [48].

## 5. Conclusions

Unveiling novel mechanistic interplay involving NRF2 role in cancer would be of benefit for the understanding of CRC progression and response to therapies. The findings in this study highlighted NRF2′s key role in neutralising the cytotoxic effects of chemotherapeutic drugs in colon cancer cells by lowering DNA damage and consequently harming p53 apoptotic activity. They also suggest that NRF2 inhibition could be a useful strategy for efficient anticancer chemotherapy and encourage the use of ZnCl_2_ supplementation to inhibit NRF2 pathway in combination therapies.

## Data Availability

Not applicable.

## Supplementary Materials

The following supporting information can be downloaded at: https://www.mdpi.com/article/10.3390/biom12030461/s1, Figure S1: Analysis of NRF2-ctr and NRF2-Cas9 clones.
