# Supplementary material for: The Impact of NRF2 Inhibition on Drug-Induced Colon Cancer Cell Death and p53 Activity: A Pilot Study
Source: Biomolecules. 2022 Mar 17;12(3):461. doi: 10.3390/biom12030461 (PMC8946796; doi:10.3390/biom12030461)
Supplement: Supplementary file 1 [file biomolecules-12-00461-s001.zip › biomolecules-1622695-supplementary.pdf]

Supplementary Figure S1

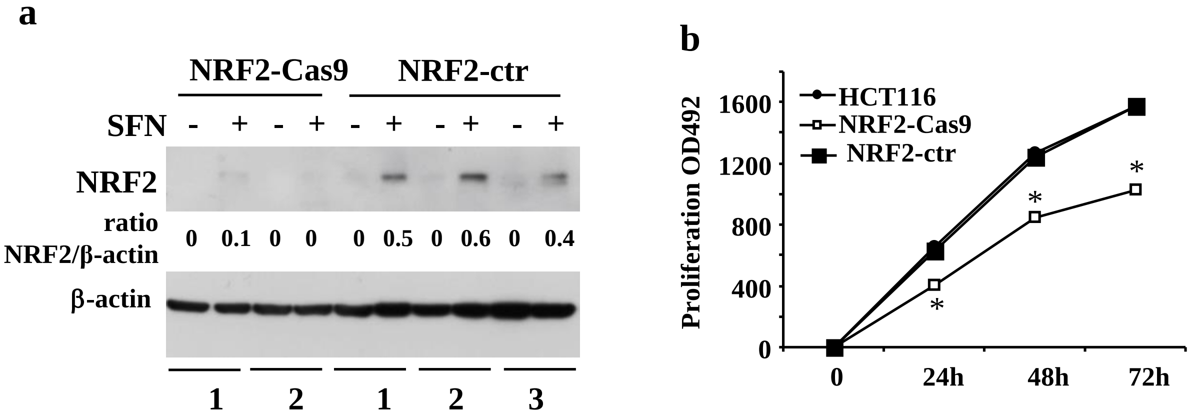

Figure S1. Analysis of NRF2 control and Cas9 clones. (**a**) NRF2-ctr (clones 1, 2, 3) and NRF2-Cas9 (clones 1, 2) were treated SFN (2 µM) for 16 h and then analysed by Western blot to detect NRF2 protein. Actin was used as protein loading control. The ratio of NRF2 levels *vs* β-actin, following densitometric analysis using ImageJ software, is shown. (**b**) NRF2-ctr (clone 2), NRF2-Cas9 (clon 2) and HCT116 cells were exposed to SFN (2 µM) for 24, 48 and 72 h, and cell viability was assessed by XTT assay. **P* ≤0.01.
